# When a Cardiac Tumor Mimics Valvular Disease: Giant Left Atrial Myxoma Causing Functional Mitral Stenosis

**DOI:** 10.7759/cureus.114317

**Published:** 2026-08-10

**Authors:** Marco Antonio Rodríguez Sánchez, Alejandro Cortés Sáinz, David Alejandro González Carrillo, Hugo Alberto Lugo Picos, Maria Guadalupe Zamora Carrasco, Diana Dely Garduño Felix, Jorlen Najera Lopez, Pedro Antonio Nieto Morga

**Affiliations:** 1 Cardiology, General Hospital of Culiacán, Autonomous University of Sinaloa, Culiacán, MEX; 2 Cardiology, Center for Research and Teaching in Health Sciences, Autonomous University of Sinaloa, Culiacán, MEX; 3 Cardiology, Civil Hospital of Culiacán, Culiacán, MEX

**Keywords:** cardiac myxoma, cardiac tumor, echocardiography, functional mitral stenosis, giant atrial myxoma

## Abstract

Cardiac myxomas are the most common primary cardiac tumors and may present with nonspecific manifestations, frequently leading to delayed diagnosis. Large left atrial myxomas can rarely mimic valvular heart disease by causing dynamic obstruction of transmitral flow.

We report the case of a 46-year-old previously healthy woman presenting with progressive exertional dyspnea and orthopnea. Transthoracic echocardiography demonstrated a giant left atrial mass measuring 69 × 34 × 31 mm attached to the interatrial septum, prolapsing into the left ventricle during diastole and causing functional mitral stenosis through a dynamic ball-valve mechanism, with a mean transmitral gradient of 9.81 mmHg. Surgical resection was successfully performed, and histopathological examination confirmed atrial myxoma. The postoperative course was uneventful with complete symptom resolution.

This case highlights the importance of considering structural intracardiac lesions in patients with unexplained heart failure symptoms and illustrates how giant atrial myxomas may mimic valvular pathology.

## Introduction

Primary cardiac tumors are rare entities, with an estimated prevalence ranging from 0.001% to 0.03%; however, approximately 85-90% are benign lesions. Among these, cardiac myxomas represent the most common histological subtype, accounting for nearly 50-75% of primary cardiac tumors in adults [1-3]. Despite their benign nature, these tumors may lead to significant morbidity and potentially life-threatening complications due to intracardiac obstruction, embolic events, or constitutional manifestations [1,4].

Cardiac myxomas occur predominantly in middle-aged women and are most commonly located in the left atrium, accounting for approximately 75-85% of cases, frequently arising from the interatrial septum near the fossa ovalis [2,5]. Clinical manifestations classically fall into three categories: obstructive cardiac symptoms, systemic embolization, and constitutional symptoms related to cytokine release [6]. The clinical presentation is highly variable and may depend on tumor size, mobility, surface characteristics, and anatomical location [7].

Large pedunculated left atrial myxomas may prolapse through the mitral valve during diastole and produce dynamic obstruction of transmitral flow through a “ball-valve” mechanism, thereby mimicking native mitral stenosis and resulting in symptoms of heart failure and pulmonary hypertension [5,8]. In this setting, functional mitral stenosis refers to dynamic obstruction of transmitral flow caused by intermittent tumor prolapse through the mitral orifice despite structurally normal mitral valve leaflets, rather than fixed anatomical narrowing of the valve. Such presentations are uncommon and may create substantial diagnostic challenges because of their nonspecific manifestations and resemblance to primary valvular disease [5].

We present the case of a giant left atrial myxoma measuring 69 × 34 × 31 mm, causing significant functional mitral stenosis through dynamic transmitral obstruction, with a mean transmitral gradient of 9.81 mmHg and progressive heart failure symptoms in an otherwise healthy middle-aged woman. The combination of a giant tumor, clinically significant dynamic obstruction, and marked symptoms despite only mild left atrial remodeling represents an uncommon presentation and provides an important educational opportunity to distinguish tumor-related functional mitral stenosis from primary mitral valve disease.

## Case presentation

A 46-year-old previously healthy woman with a past surgical history of cholecystectomy and two uncomplicated cesarean sections presented with a five-month history of progressively worsening exertional dyspnea. Initially, symptoms occurred only during strenuous physical activity; however, they gradually progressed to moderate and eventually minimal exertion, resulting in a marked decline in functional capacity and significant limitation of daily activities. The patient also reported progressively worsening orthopnea. She denied chest pain, palpitations, syncope, constitutional symptoms such as fever or unintentional weight loss, and any history suggestive of embolic events.

The progressive development of heart failure-like symptoms in an otherwise healthy middle-aged woman without significant cardiovascular risk factors raised suspicion for an underlying structural cardiac abnormality, prompting further diagnostic evaluation.

On presentation, the patient was hemodynamically stable and in no acute distress. Cardiovascular examination revealed no jugular venous distention, while pulmonary auscultation demonstrated preserved bilateral breath sounds without crackles or wheezing. Cardiac auscultation identified a regular rhythm with a systolic murmur best heard at the mitral area without radiation or additional heart sounds. Peripheral pulses were symmetric and preserved, capillary refill time was normal, and no peripheral edema was observed.

At presentation, the patient was in New York Heart Association (NYHA) functional class II-III. Admission vital signs were as follows: blood pressure 115/75 mmHg, heart rate 90 beats/min, respiratory rate 15 breaths/min, oxygen saturation 96% on room air, and body temperature 36.0°C.

Initial laboratory investigations revealed mild normocytic anemia (hemoglobin 9.8 g/dL), preserved renal function (creatinine 0.43 mg/dL), and elevated B-type natriuretic peptide (BNP) levels (548 pg/mL), suggesting increased intracardiac filling pressures. Electrolytes and coagulation parameters were within normal limits (Table 1).

**Table 1:** Initial laboratory findings Initial laboratory findings demonstrating mild normocytic anemia and elevated B-type Natriuretic Peptide (BNP) levels suggestive of increased intracardiac filling pressures.

Laboratory Parameter	Result	Reference Range
Prothrombin Time (PT), sec	15	11–15 sec
International Normalized Ratio (INR)	1.17	0.8–1.2
Activated Partial Thromboplastin Time (aPTT), sec	33	25–35 sec
Triglycerides, mg/dL	37	<150 mg/dL
Low-density Lipoprotein (LDL), mg/dL	49	<100 mg/dL
Hemoglobin A1c (HbA1c), %	6.5	<5.7%
Glucose, mg/dL	102	70–99 mg/dL
Urea, mg/dL	22.5	15–40 mg/dL
Creatinine, mg/dL	0.43	0.5–1.1 mg/dL
B-type Natriuretic Peptide (BNP), pg/mL	548	<100 pg/mL
Sodium (Na), mmol/L	138	135–145 mmol/L
Potassium (K), mmol/L	4.1	3.5–5.0 mmol/L
Chloride (Cl), mmol/L	104	98–107 mmol/L
Phosphorus (P), mg/dL	3.3	2.5–4.5 mg/dL
Hemoglobin (Hb), g/dL	9.8	12–16 g/dL
Hematocrit (Hct), %	31	36–46%
Platelet count, ×10³/µL	329	150–450 ×10³/µL
White blood cell count, ×10³/µL	6.52	4–10 ×10³/µL

**Figure 1:**
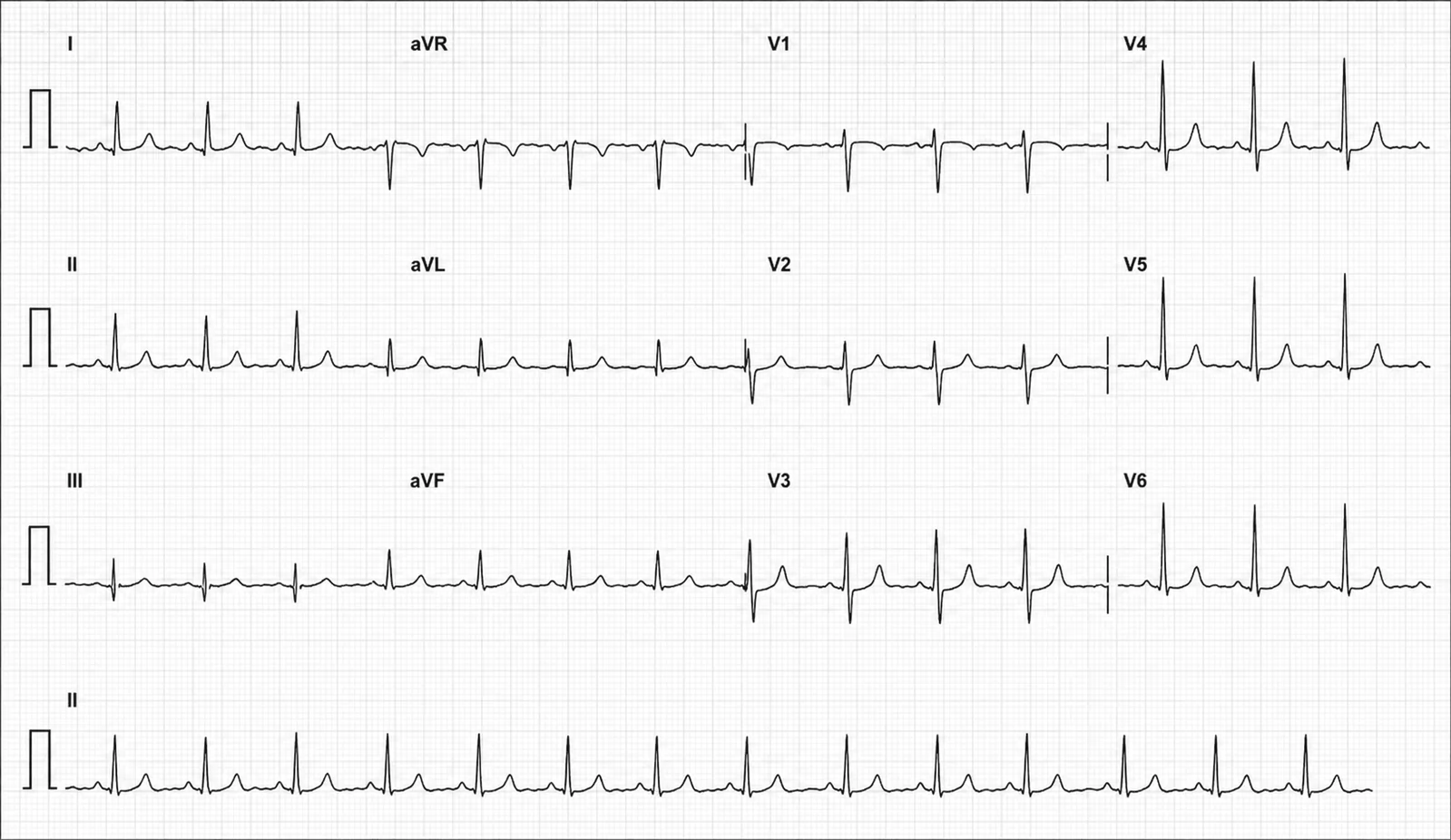
Admission electrocardiogram Twelve-lead electrocardiogram demonstrating normal sinus rhythm with preserved R-wave progression across the precordial leads and no evidence of conduction abnormalities, chamber hypertrophy, or acute ischemic changes.

Electrocardiography demonstrated sinus rhythm at 85 beats/min with a normal axis and intervals, preserved R-wave progression across the precordial leads, and no evidence of chamber hypertrophy, conduction abnormalities, or acute ischemic changes (Figure 1).

**Figure 2:**
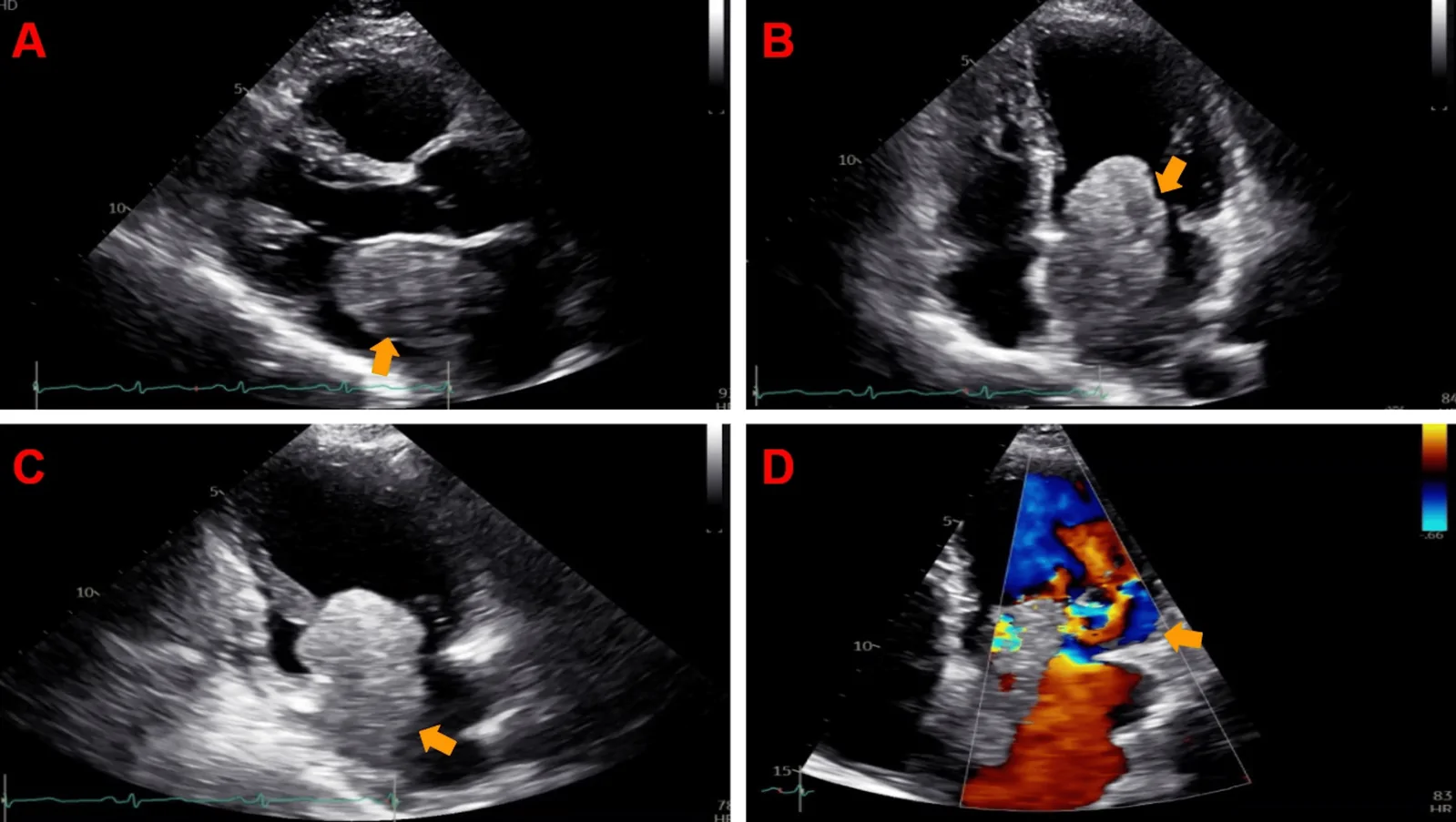
Transthoracic echocardiographic assessment of a giant left atrial myxoma (A) Parasternal long-axis view demonstrating a large, heterogeneous, highly mobile left atrial mass (orange arrow). (B) Apical four-chamber view showing diastolic prolapse of the mass (orange arrow) through the mitral valve into the left ventricle. (C) Apical four-chamber view demonstrating the pedunculated mass (orange arrow) attached to the interatrial septum, occupying nearly half of the left atrial cavity. (D) Color Doppler imaging demonstrating dynamic transmitral flow obstruction caused by the prolapsing mass (orange arrow), producing functional mitral stenosis through a "ball-valve" mechanism.

**Figure 3:**
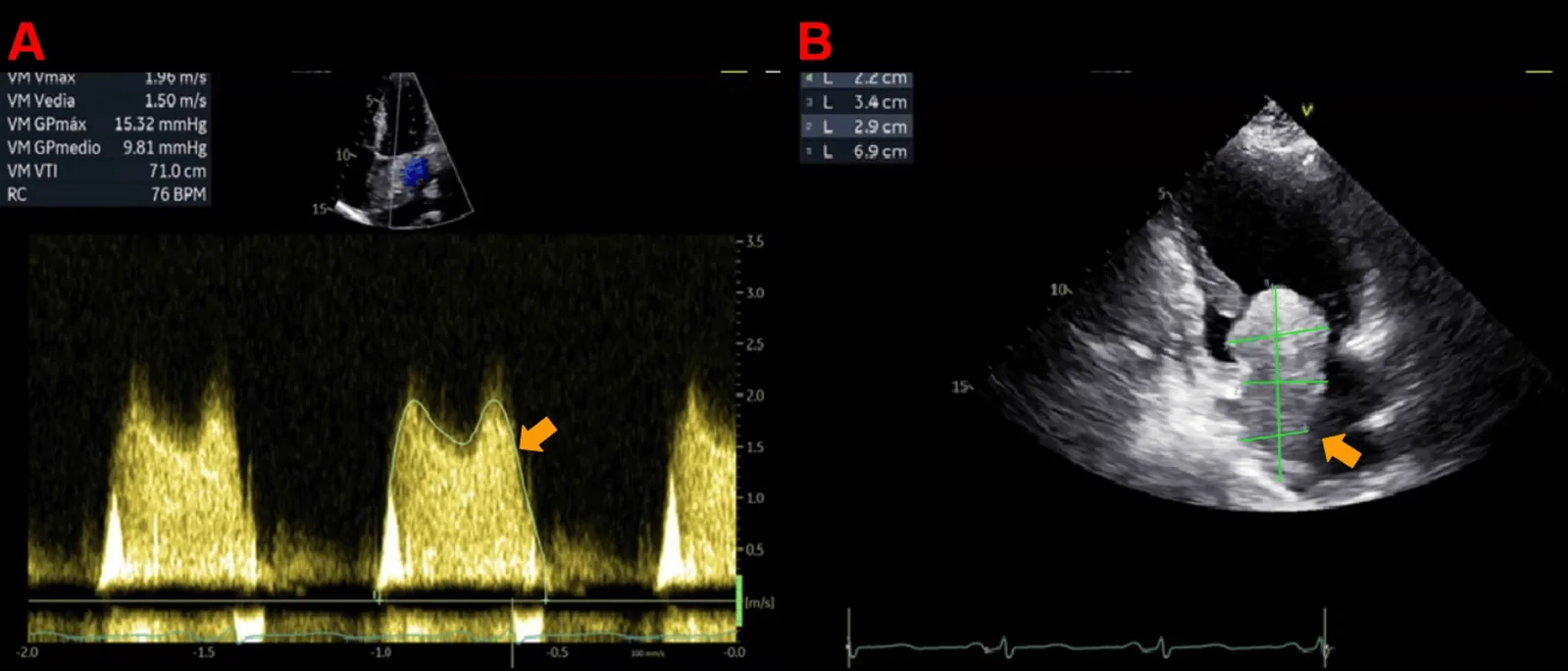
Echocardiographic characterization of the left atrial mass (A) Continuous-wave Doppler across the mitral valve demonstrating dynamic transmitral inflow obstruction caused by a "ball-valve" mechanism due to diastolic prolapse of the left atrial mass (orange arrow), generating a mean transmitral pressure gradient of 9.81 mmHg, consistent with hemodynamically significant functional mitral stenosis. (B) Apical four-chamber view demonstrating a large left atrial mass attached to the interatrial septum (orange arrow). Multiplanar measurements show maximum dimensions of 69 × 34 × 31 mm, consistent with a giant left atrial myxoma.

**Figure 4:**
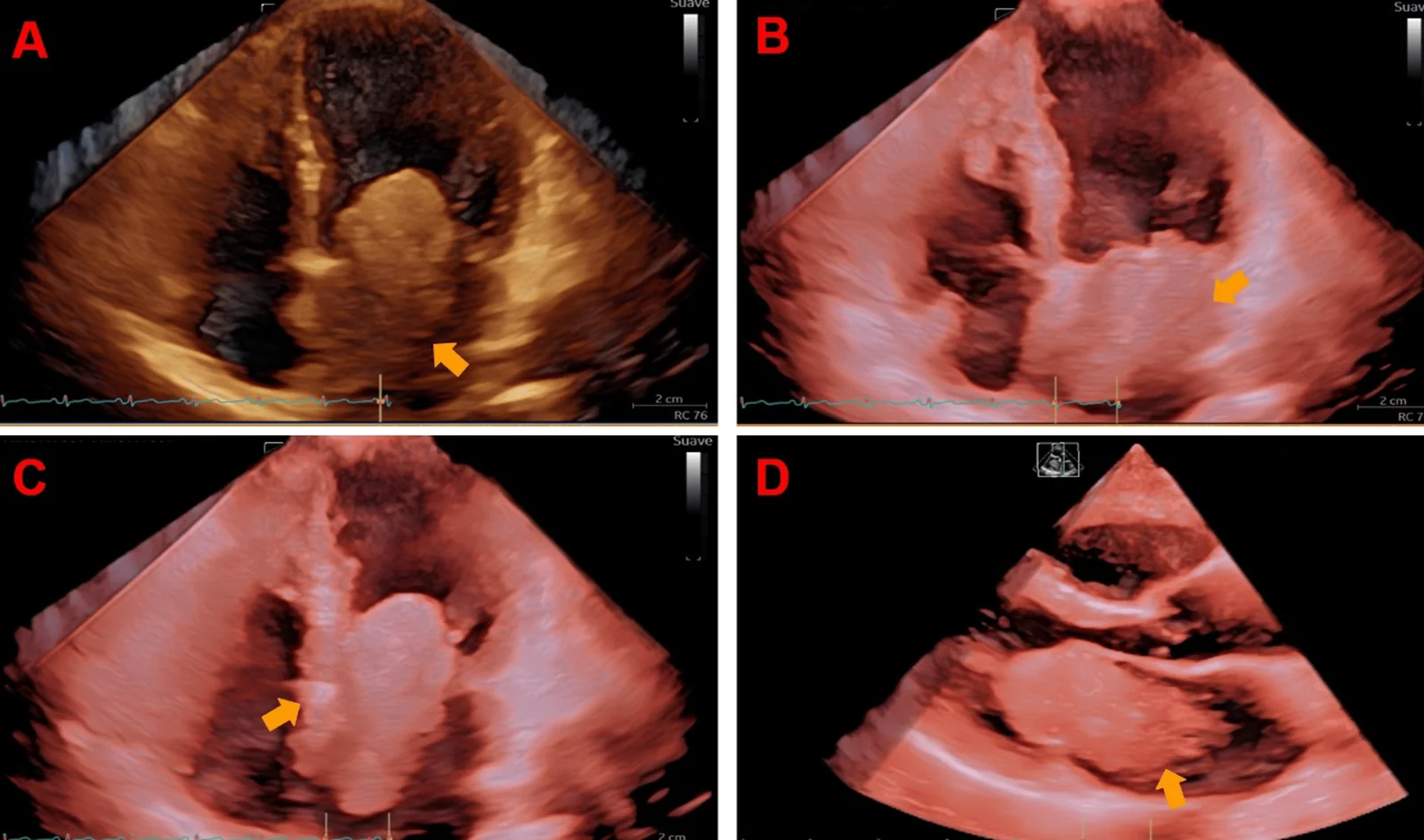
Transthoracic echocardiogram 3D image Three-dimensional transthoracic echocardiography demonstrating a large, highly mobile left atrial mass attached to the interatrial septum, occupying more than 50% of the left atrial cavity. (A–C) Three-dimensional apical views illustrating the pedunculated mass (orange arrows), its broad spatial extension, and dynamic mobility within the left atrium. (D) Parasternal long-axis three-dimensional view confirming the large intracavitary mass arising from the interatrial septum and projecting toward the mitral valve, consistent with a left atrial myxoma.

Given the patient's progressive symptoms, physical examination findings, and BNP level disproportionately elevated for her age and absence of major comorbidities, transthoracic echocardiography was performed. This revealed a large, mobile, heterogeneous intracardiac mass arising from the left atrium, measuring 69 × 34 × 31 mm, highly suggestive of a cardiac myxoma. The lesion was pedunculated and originated from the interatrial septum, occupying approximately 50% of the left atrial cavity. During diastole, the mass prolapsed through the structurally preserved mitral valve into the left ventricle, producing significant dynamic obstruction of transmitral inflow through a “ball-valve” mechanism and resulting in functional rather than anatomical mitral stenosis (Figures 2-4).

Left ventricular systolic function was preserved, with an ejection fraction of 66% and no regional wall motion abnormalities. Mild left atrial enlargement was observed, with a left atrial volume index of 39 mL/m². Continuous-wave Doppler demonstrated a mean transmitral gradient of 9.81 mmHg, while planimetry showed a mitral valve area of 4.6 cm², supporting the presence of dynamic tumor-related obstruction rather than intrinsic mitral valve stenosis. Echocardiographic findings suggested an intermediate probability of pulmonary hypertension, with an estimated pulmonary artery systolic pressure of 43 mmHg and a peak tricuspid regurgitation velocity of 3.1 m/s. Right ventricular dimensions and systolic function were preserved, with a tricuspid annular plane systolic excursion (TAPSE) of 24 mm.

**Figure 5:**
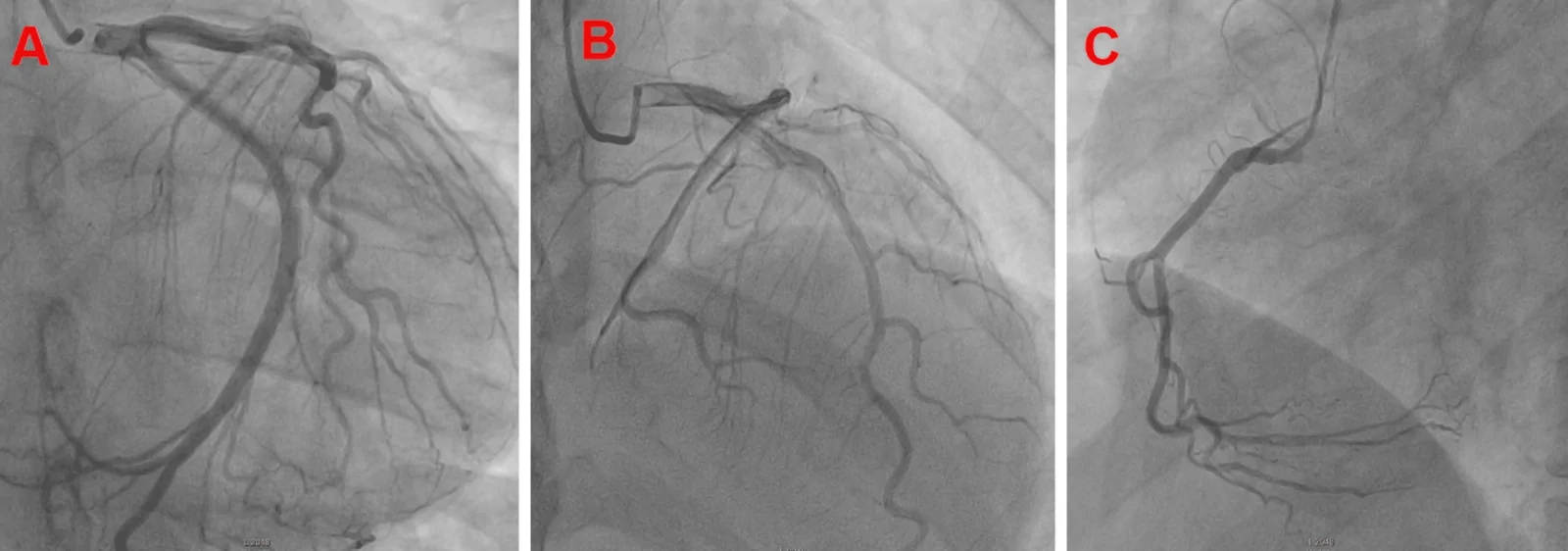
Coronary angiography Preoperative coronary angiography demonstrating angiographically normal coronary arteries without evidence of obstructive coronary artery disease. (A) Left coronary angiogram showing a patent left main coronary artery with unobstructed bifurcation into the left anterior descending and left circumflex arteries. (B) Alternative projection of the left coronary system confirming the absence of significant luminal stenosis in the left anterior descending and left circumflex arteries. (C) Right coronary angiogram demonstrating a patent right coronary artery without angiographically significant stenosis.

Given the patient's age and clinical presentation, invasive coronary angiography was performed as part of the preoperative assessment to evaluate coronary anatomy and exclude concomitant ischemic heart disease. Coronary angiography demonstrated no significant obstructive coronary artery disease (Figure 5).

Because transthoracic echocardiography provided excellent visualization of the mass, its attachment site, mobility, and hemodynamic consequences, additional imaging with transesophageal echocardiography, cardiac CT, or cardiac MRI was not considered necessary for diagnosis or surgical planning. 

The case was subsequently discussed by the institutional Heart Team, and surgical resection of the left atrial mass was recommended. Intraoperative findings revealed a giant myxomatous mass measuring approximately 70 × 50 × 40 mm attached to the interatrial septum and extending toward the lateral wall of the left atrium. Cardiopulmonary bypass time was 60 minutes, with an aortic cross-clamp time of 40 minutes. The mitral and tricuspid valves were structurally preserved and functionally normal. Estimated intraoperative blood loss was approximately 800 mL, without major procedural complications. 

**Figure 6:**
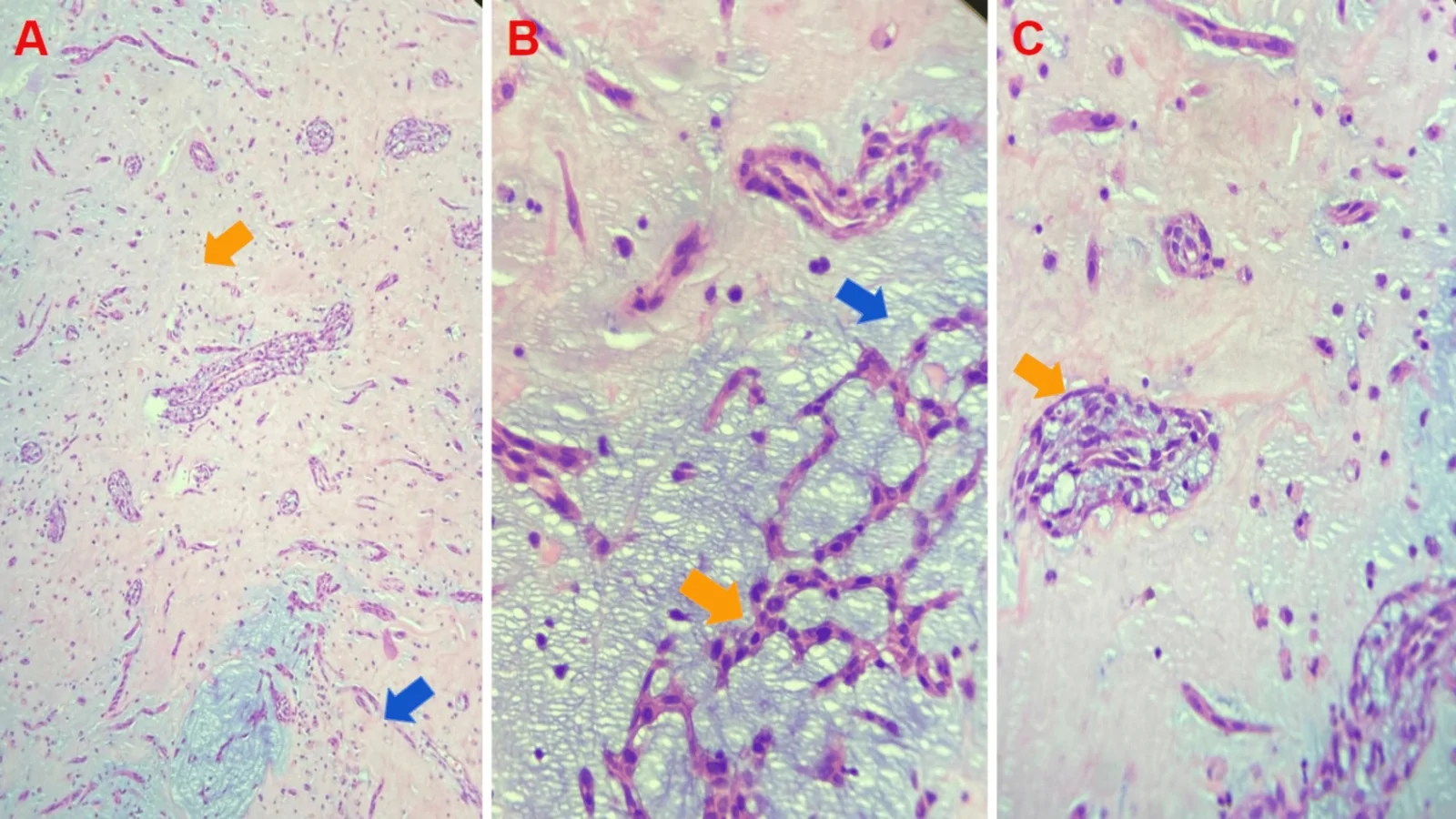
Histopathological examination of the resected cardiac mass (hematoxylin and eosin stain) (A) Low-power photomicrograph demonstrating abundant myxoid stroma (orange arrow) containing scattered thin-walled vascular channels (blue arrow). (B) Higher-power view showing stellate and polygonal myxoma cells (orange arrow) embedded within a prominent myxoid matrix (blue arrow). (C) High-power view highlighting the characteristic perivascular arrangement of myxoma cells (orange arrow) surrounding delicate capillary-sized vessels, a classic histopathological feature of cardiac myxoma.

Histopathological examination of the resected specimen demonstrated a hypocellular neoplasm composed of abundant myxoid stroma containing scattered stellate and spindle-shaped cells arranged around delicate vascular structures, findings consistent with a cardiac myxoma (Figure 6). 

The postoperative course was uneventful. The patient remained hospitalized for five days following surgery, with pleural and mediastinal drains successfully removed on postoperative day three. Follow-up transthoracic echocardiography demonstrated complete resection of the intracardiac mass, without evidence of pericardial effusion or significant residual valvular abnormalities.

At outpatient follow-up, the patient remained asymptomatic (NYHA functional class I). Clinical and transthoracic echocardiographic follow-up was performed monthly during the first six postoperative months and every three months thereafter, demonstrating no evidence of tumor recurrence or residual functional mitral obstruction.

## Discussion

Although the epidemiology and typical clinical manifestations of cardiac myxomas are well established, their presentation may vary considerably according to tumor size, mobility, and relationship with adjacent cardiac structures. The present case illustrates an uncommon hemodynamic manifestation in which a giant left atrial myxoma produced clinically significant functional mitral stenosis through a dynamic ball-valve mechanism, closely mimicking primary mitral valve disease. 

Obstructive manifestations result from mechanical interference with intracardiac blood flow and may mimic various forms of structural heart disease. Large pedunculated left atrial myxomas can intermittently prolapse through the mitral valve during diastole, generating dynamic obstruction of transmitral inflow through a “ball-valve” mechanism [5,8]. This phenomenon may produce hemodynamic findings similar to those observed in native mitral stenosis, including elevated left atrial pressures, pulmonary venous congestion, pulmonary hypertension, and progressive exertional dyspnea [8,9]. In the present case, the lesion occupied nearly half of the left atrial cavity and generated a mean transmitral gradient of 9.81 mmHg, supporting the presence of significant functional mitral stenosis secondary to dynamic obstruction.

Only a limited number of published reports have described giant left atrial myxomas presenting with clinically significant functional mitral stenosis caused by a dynamic ball-valve mechanism. Similar to previously reported cases (Table 2), our patient presented with progressive heart failure symptoms despite structurally normal mitral valve leaflets, and transthoracic echocardiography demonstrated dynamic transmitral obstruction requiring prompt surgical excision [10,11,12]. However, our case is distinguished by the comprehensive hemodynamic characterization obtained with transthoracic echocardiography alone, including detailed Doppler assessment of the functional obstruction, which provided sufficient information for diagnosis and surgical planning without the need for additional imaging modalities. 

**Table 2:** Comparison of clinical, echocardiographic, and surgical findings in previously reported cases of giant left atrial myxoma causing functional mitral stenosis and the present case Data are presented as reported in the original publications. The present case is characterized by a giant left atrial myxoma causing dynamic transmitral obstruction despite structurally preserved mitral valve leaflets. LA, left atrial; PASP, pulmonary artery systolic pressure; LAVI, left atrial volume index; TTE, transthoracic echocardiography; TEE, transesophageal echocardiography; NYHA, New York Heart Association.

Variable	Bozkurt et al. (2026) [11]	Almimar et al. (2026) [12]	Present case
Tumor size	Large LA myxoma	Giant (>8 cm)	69 × 34 × 31 mm
Mean transmitral gradient	Increased	Dynamic obstruction	9.81 mmHg
Pulmonary hypertension	Present	Mild	PASP 43 mmHg
Left atrial remodeling	Mild	Mild	LAVI 39 mL/m²
Embolic events	No	No	No
Imaging	TTE ± TEE	Multimodality	TTE alone
Surgical outcome	Complete recovery	Complete recovery	Complete recovery, NYHA I

Although mild normocytic anemia (hemoglobin 9.8 g/dL) may have contributed to reduced exercise tolerance, it was unlikely to represent the primary cause of the patient's symptoms. The progressive nature of dyspnea and orthopnea, elevated BNP levels, significant dynamic transmitral obstruction, and complete symptom resolution following surgical excision strongly support the left atrial myxoma as the principal mechanism underlying the patient's heart failure symptoms. 

A mean transmitral gradient of 9.81 mmHg is hemodynamically comparable to that observed in severe rheumatic mitral stenosis, despite the absence of intrinsic valvular pathology. Unlike rheumatic mitral stenosis, in which the elevated gradient results from fixed leaflet obstruction and reduced valve area, the present case demonstrated dynamic obstruction produced by intermittent prolapse of the tumor through the mitral orifice. This distinction has important therapeutic implications because definitive treatment requires tumor excision rather than intervention on the mitral valve itself. 

Interestingly, our patient lacked several of the common findings associated with advanced mitral stenosis despite significant obstruction. No atrial fibrillation, severe pulmonary hypertension, or substantial left atrial enlargement were observed. This finding may be explained by the relatively short duration of symptoms and the dynamic rather than fixed nature of the obstruction. Unlike rheumatic mitral stenosis, where progressive structural narrowing results in chronic pressure overload, dynamic obstruction caused by myxomas may produce substantial hemodynamic compromise while inducing less pronounced anatomical remodeling [7].

Echocardiography remains the cornerstone of diagnosis for cardiac myxomas because of its wide availability, noninvasive nature, and excellent diagnostic performance. Transthoracic echocardiography has a reported sensitivity exceeding 90%, while transesophageal echocardiography may further improve anatomical characterization and surgical planning [7,9]. Echocardiographic assessment not only allows identification of tumor size and attachment site but also provides essential hemodynamic information, including valve involvement, intracardiac obstruction, and secondary pulmonary hypertension. In our patient, echocardiography was crucial for identifying the pedunculated attachment to the interatrial septum and the dynamic transmitral obstruction responsible for symptom development.

Prompt surgical excision remains the treatment of choice after diagnosis because of the risk of embolic complications, acute obstruction, and sudden cardiac death [1,5]. Surgical outcomes are generally excellent, with operative mortality rates below 5% and recurrence rates reported between 1% and 5% in sporadic cases [10]. Complete excision with removal of the implantation site is recommended to minimize recurrence risk. Our patient underwent successful surgical resection with complete symptom resolution and no evidence of residual obstruction on postoperative echocardiographic evaluation.

Recurrence after surgical excision is uncommon in sporadic cardiac myxomas but may occur because of incomplete excision, multicentric tumor origin, or familial syndromes such as Carney complex. Our patient had no clinical or family history suggestive of a hereditary syndrome, and complete surgical excision was achieved, making the estimated recurrence risk low. Nevertheless, periodic echocardiographic surveillance remains recommended. 

This case highlights an unusual presentation of left atrial myxoma manifesting predominantly as progressive heart failure symptoms due to significant dynamic transmitral obstruction. It further emphasizes the importance of considering intracardiac tumors in the differential diagnosis of unexplained dyspnea and apparent valvular heart disease, particularly in patients without conventional cardiovascular risk factors.

Recognizing functional mitral stenosis secondary to a left atrial myxoma is clinically important because its management differs fundamentally from that of primary valvular mitral stenosis. Failure to identify the underlying intracardiac mass may delay definitive surgical treatment, increasing the risk of embolic events, progressive heart failure, acute hemodynamic deterioration, or sudden cardiac death. Comprehensive echocardiographic evaluation is therefore essential to distinguish dynamic obstruction caused by a cardiac tumor from intrinsic mitral valve disease and to ensure timely referral for surgical excision. 

This case report has several limitations. First, as a single-case report, its findings cannot be generalized to all patients with giant left atrial myxomas causing functional mitral stenosis. Second, although postoperative follow-up demonstrated no evidence of recurrence, the duration of follow-up remains relatively limited for assessing long-term outcomes. Finally, additional imaging modalities such as transesophageal echocardiography, cardiac computed tomography, or cardiac magnetic resonance imaging were not performed because transthoracic echocardiography provided sufficient anatomical and hemodynamic information for diagnosis and surgical planning.

## Conclusions

Giant left atrial myxomas represent an uncommon but important cause of intracardiac obstruction and may closely mimic primary valvular heart disease. This case illustrates how a large pedunculated atrial myxoma can produce significant functional mitral stenosis through a dynamic ball-valve mechanism, leading to progressive heart failure symptoms despite the absence of major cardiovascular risk factors. In this patient, early transthoracic echocardiographic assessment enabled recognition of the intracardiac mass, characterization of the dynamic transmitral obstruction, and timely surgical referral, with complete resolution of symptoms following resection. The patient remained asymptomatic in NYHA functional class I during serial clinical and echocardiographic follow-up, performed monthly during the first six postoperative months and every three months thereafter. Although the findings of a single case cannot be generalized, this presentation highlights the potential diagnostic value of echocardiography in patients with unexplained heart failure symptoms and suspected intracardiac obstruction. The educational value of this case lies in demonstrating that giant left atrial myxomas, although rare, can produce functional mitral stenosis through a dynamic ball-valve mechanism and closely simulate primary mitral valve disease. Following complete surgical excision, periodic clinical and echocardiographic follow-up is important to monitor for the rare possibility of tumor recurrence. 
